# Association of Concomitant Amlodipine Use With Serum Olanzapine Concentrations and Clinical Outcomes in Patients With Schizophrenia

**DOI:** 10.1002/hup.70062

**Published:** 2026-09-11

**Authors:** Xiaoxiao Chen, Jing Ma, Xiaolei Xuan, Mao Huang, Xue Yang, Baorong Huang

**Affiliations:** ^1^ Department of Clinical Laboratory Wuchang Hospital Affiliated to Wuhan University of Science and Technology Wuhan China; ^2^ Hubei Province Key Laboratory of Occupational Hazard Identification and Control Wuhan University of Science and Technology Wuhan China; ^3^ Department of Clinical Laboratory Third Affiliated Hospital of Henan University of Traditional Chinese Medicine Zhengzhou China

**Keywords:** amlodipine, olanzapine, retrospective study, schizophrenia, therapeutic drug monitoring

## Abstract

**Objective:**

To examine whether concomitant amlodipine use was associated with steady‐state serum olanzapine concentration and clinical outcomes in patients with schizophrenia.

**Methods:**

This retrospective study included 45 inpatients with schizophrenia and hypertension who received olanzapine 10 mg/day plus amlodipine 5 mg/day (experimental group) and 52 inpatients who received olanzapine 10 mg/day alone (control group). PANSS and SDSS scores were assessed at baseline and week 7, and recorded adverse events were evaluated at week 9.

**Results:**

Mean serum olanzapine concentration was lower in the combination group than in the olanzapine‐alone group (40.56 ± 18.86 vs. 50.23 ± 23.41 ng/mL; unadjusted *p* = 0.027; age‐adjusted *p* = 0.032) (Figure 1). Improvements in positive symptoms, negative symptoms, and SDSS scores were smaller in the combination group, whereas PANSS total and general psychopathology changes did not differ. Serum olanzapine concentration was not significantly correlated with any PANSS or SDSS change. Recorded adverse‐event rates did not differ significantly.

**Conclusions:**

Concomitant amlodipine use was associated with lower serum olanzapine concentration and smaller improvements in selected clinical domains. The individual‐level analyses did not establish a concentration‐response relationship, and the retrospective design precludes causal inference. Haemodynamic safety could not be evaluated because complete standardised blood‐pressure data were unavailable. These findings suggest that closer monitoring of clinical response and serum olanzapine concentrations may be considered when amlodipine is co‐prescribed, particularly in patients with suboptimal treatment response.

## 1 Introduction

Schizophrenia is a chronic mental disorder associated with substantial personal, family, and societal burden (Velligan and Rao 2023). People with schizophrenia also experience marked excess cardiovascular morbidity and premature mortality, driven by an interaction among illness‐related factors, smoking, cardiometabolic risk, antipsychotic exposure, and inequalities in physical health care (Correll et al. 2017; Murphy and Daumit 2023; Polcwiartek et al. 2024). Hypertension is therefore a common and clinically important comorbidity in this population (Sudarshan and Cheung 2023). Olanzapine remains widely used because of its efficacy across several symptom domains, although its metabolic adverse‐effect profile and substantial pharmacokinetic variability complicate long‐term treatment (Kolli et al. 2023; Sampogna et al. 2023).

Olanzapine disposition is influenced by dose, age, sex, body weight, smoking, genetic variation, sampling time, and concomitant medications (An et al. 2021; Ansermot et al. 2024; Horvat et al. 2023). CYP1A2 is an important oxidative pathway, whereas CYP3A4 contributes to a lesser extent; recent pharmacogenetic guidance also emphasizes that the clinical relevance of CYP variation differs among antipsychotics and should not be inferred without drug‐specific evidence (Beunk et al. 2024; Zhang et al. 2024). Cigarette smoking is particularly relevant because induction of CYP1A2 can lower olanzapine exposure (Horvat et al. 2023; Zanni et al. 2025). Amlodipine is a dihydropyridine calcium‐channel blocker metabolised mainly by CYP3A4/5 and has been described as a weak CYP3A4 inhibitor (Curran 2010; Zhu et al. 2014). The most widely recognised concern when olanzapine and amlodipine are co‐administered is pharmacodynamic, namely additive blood‐pressure lowering. A clinically established pharmacokinetic interaction has not been demonstrated, and weak CYP3A4 inhibition would not readily predict lower olanzapine exposure.

Evidence concerning calcium‐channel blockers and antipsychotic outcomes remains limited. In an analysis of CATIE‐Sz data, concomitant calcium‐channel blocker use was associated with change in Clinical Global Impression‐Severity scores but not with PANSS total scores or all‐cause antipsychotic discontinuation (Van Dyke and Thomas 2018). That study evaluated clinical outcomes rather than a specific pharmacokinetic interaction between amlodipine and olanzapine. More recent literature supports therapeutic drug monitoring of olanzapine when non‐response, adverse effects, adherence concerns, or drug interactions are clinically plausible, but also highlights the need to interpret concentrations in the context of patient‐level covariates (Ansermot et al. 2024; Bhavsar et al. 2024; Hadjoudj et al. 2024; Wesner et al. 2023).

Because olanzapine and amlodipine are frequently co‐prescribed in clinical practice, we conducted an exploratory retrospective comparison to determine whether concomitant amlodipine use was associated with steady‐state olanzapine concentration, longitudinal PANSS and SDSS changes, and recorded adverse events. We also examined whether individual olanzapine concentrations were associated with clinical improvement. As hypertension status and amlodipine exposure were inseparable in this dataset, the study was designed to identify associations rather than establish a causal drug‐drug interaction.

## 2 Methods

### 2.1 Study Design and Participants

This single‐centre retrospective study screened consecutive patients hospitalised in the Department of Psychiatry, Wuhan Wuchang Hospital, from January 2023 to December 2025.

Inclusion criteria for the combination group, also known as the experimental group (schizophrenia with hypertension, *n* = 45) were: (1) meeting ICD‐10 criteria for schizophrenia; (2) comorbid essential hypertension adequately controlled with amlodipine; (3) age ≥ 40 years; (4) no olanzapine use before the index treatment period or an olanzapine washout of at least 2 weeks; and (5) treatment with olanzapine 10 mg/day plus amlodipine 5 mg/day.

Inclusion criteria for the olanzapine‐alone group, also known as the control group (schizophrenia without hypertension, *n* = 52) were: (1) meeting ICD‐10 criteria for schizophrenia; (2) age ≥ 40 years; (3) no olanzapine use before the index treatment period or an olanzapine washout of at least 2 weeks; and (4) treatment with olanzapine 10 mg/day alone. The cohort was not necessarily antipsychotic‐naive; the eligibility criterion referred specifically to olanzapine exposure, and complete prior exposure to other antipsychotics was not available retrospectively.

Exclusion criteria for both groups were severe unstable physical illness, liver or renal failure, other major psychiatric disorders, epilepsy or traumatic brain injury likely to affect assessment, pregnancy or lactation, known hypersensitivity to olanzapine or amlodipine, and alcohol or substance dependence.

### 2.2 Baseline Data Collection

Demographic and clinical data extracted from medical records included age, sex, body weight, alanine aminotransferase, aspartate aminotransferase, current daily smoking during the 6 months before admission, family history of schizophrenia, and baseline clinical scores. Illness duration, smoking intensity, complete prior antipsychotic exposure, adherence, and comprehensive concomitant‐medication histories were not consistently available. Routine blood‐pressure measurements were incomplete and were obtained at variable, non‐standardised time points; orthostatic measurements and hypotension‐related symptoms were not systematically documented. Consequently, reliable baseline‐to‐follow‐up blood‐pressure analyses could not be performed.

### 2.3 Drug Administration and Therapeutic Drug Monitoring

All patients received oral olanzapine 10 mg once daily at bedtime. Patients in the combination group additionally received oral amlodipine besylate 5 mg once daily in the morning.

After 2 weeks of treatment, morning predose serum samples were obtained for steady‐state olanzapine measurement using an AB Sciex 4500MD liquid chromatography‐tandem mass spectrometry system. Sample preparation, solvent preparation, calibration, and quality‐control procedures followed the commercial reagent instructions used by the laboratory. Briefly, 50 μL of serum, calibrator, or quality‐control material was combined with 50 μL of internal‐standard working solution, mixed at 1200 rpm for 5 min, and centrifuged at 4000 rpm for 5 min. Forty microlitres of supernatant was diluted with 600 μL of 0.1% formic acid in water, mixed and centrifuged again, and 5 μL was injected. Seven calibration levels and low‐ and high‐concentration quality controls were used, with 1/x^2^ weighted least‐squares calibration.

The laboratory performance verification for olanzapine showed linearity from 5 to 300 ng/mL, acceptable within‐run and between‐run precision, recoveries of 100.7% and 107.4%, and a lower limit of quantification of 5 ng/mL. Linearity and precision results are presented in Tables S1–S3; recovery, lower limit of quantification, and carryover results are shown in Table S4; interference results are shown in Table S5; and matrix‐effect and dilution‐integrity results are shown in Table S6 in Supporting Information S1.

### 2.4 Efficacy and Safety Assessment

Clinical outcomes were assessed by trained psychiatrists using the Positive and Negative Syndrome Scale (PANSS) and the Social Disability Screening Schedule (SDSS) at baseline and week 7. The PANSS total score and the positive‐symptom, negative‐symptom, and general psychopathology domain scores recorded in the electronic medical record were extracted separately. Change scores were calculated for each patient as baseline minus week 7, so larger positive values indicated greater improvement. The PANSS total score and the positive‐symptom, negative‐symptom, and general psychopathology domain scores routinely recorded in the electronic medical records were extracted as separate clinical variables. In the local clinical documentation system, these three recorded domain scores did not constitute an exhaustive decomposition of the PANSS total score, because additional rated items contributed to the recorded total score. Therefore, the domain scores were analysed separately and were not summed to reconstruct or verify the PANSS total score.

Safety was assessed at week 9 using the available medical‐record and Rating Scale for Extrapyramidal Side Effects documentation. Extrapyramidal symptoms and other commonly recorded adverse events were coded as present or absent. The analytical dataset contained only a binary indicator of any recorded adverse event; event‐specific and haemodynamic outcomes could not be analysed reliably.

### 2.5 Statistical Analysis

Data were analysed using SPSS version 21.0 (IBM Corp., Armonk, NY, USA). Continuous variables are presented as mean ± standard deviation when approximately normally distributed and as median (interquartile range) when non‐normally distributed. Categorical variables are presented as number (percentage). Between‐group comparisons used the independent‐samples *t* test or Welch test for approximately normal variables, the Mann‐Whitney *U* test for non‐normal variables, and the χ^2^ test or Fisher exact test for categorical variables.

The unadjusted between‐group comparison of olanzapine concentration was treated as the primary analysis. Because age differed between groups and may influence olanzapine pharmacokinetics, an age‐adjusted ANCOVA was retained as a sensitivity analysis. Clinical change scores were compared using ANCOVA with age and the corresponding baseline score as covariates. Homogeneity of regression slopes was evaluated, and heteroskedasticity‐robust sensitivity analyses produced materially similar conclusions.

Associations between steady‐state olanzapine concentration and PANSS and SDSS change scores were assessed using two‐sided Spearman rank correlations because the clinical outcome distributions were non‐normal. Recorded adverse‐event occurrence was analysed using binary logistic regression with age as a covariate to estimate odds ratios and 95% confidence intervals. The mean ± SD in the figure is shown for descriptive purposes only; inference is primarily based on adjusted models.

All tests were two‐sided, and *p* < 0.05 was considered statistically significant. No adjustment for multiple comparisons was applied because the clinical and correlation analyses were exploratory. Findings from secondary symptom‐domain analyses were therefore interpreted cautiously.

## 3 Results

### 3.1 Baseline Characteristics

Baseline characteristics are summarised in Table 1. The combination group was older than the olanzapine‐alone group (61.71 ± 11.09 vs. 57.33 ± 8.61 years, *p* = 0.031). Sex, smoking history, family history, liver enzymes, body weight, and baseline PANSS and SDSS scores did not differ significantly between groups.

**TABLE 1:** Baseline characteristics of the experimental and control groups.

Characteristic	Experimental group (*n* = 45)	Control group (*n* = 52)	Statistic	*p* value
Age (years, mean ± SD)	61.71 ± 11.09	57.33 ± 8.61	*t* = 2.189	*p* = 0.031*
Male sex, *n* (%)	25 (55.56)	32 (61.54)	*χ* ^2^ = 0.356	*p* = 0.551
Smoking history, *n* (%)	8 (17.78)	9 (17.31)	*χ* ^2^ = 0.004	*p* = 0.952
Family history, *n* (%)	4 (8.89)	6 (11.54)	Fisher	*p* = 0.748
ALT, median (IQR)	16.4 (11.1–22.3)	17.0 (13.8–29.9)	Mann‐whitney U	*p* = 0.135
AST, median (IQR)	18.6 (14.6–23.7)	21.2 (17.0–25.0)	Mann‐whitney U	*p* = 0.072
Body weight kg (IQR)	66 (59.8–73.0)	70.5 (58.3–72.1)	Mann‐whitney U	*p* = 0.121
Baseline PANSS total, median (IQR)	126.0 (115.0, 128.0)	124.0 (116.5, 128.0)	Mann‐whitney U	*p* = 0.391
Baseline PANSS positive, median (IQR)	32.0 (32.0, 33.0)	32.0 (32.0, 35.8)	Mann‐whitney U	*p* = 0.947
Baseline PANSS negative, median (IQR)	35.0 (30.5, 35.0)	35.0 (29.0, 35.0)	Mann‐whitney U	*p* = 0.967
Baseline PANSS general psychopathology, median (IQR)	54.0 (48.0, 56.0)	56.0 (51.0, 56.0)	Mann‐whitney U	*p* = 0.525
Baseline SDSS, median (IQR)	16.0 (15.5, 16.0)	16.0 (14.0,16.0)	Mann‐whitney U	*p* = 0.581

Abbreviations: ALT, alanine aminotransferase (U/L); AST, aspartate aminotransferase (U/L); IQR, interquartile range; SD, standard deviation.

*p < 0.05 denotes a statistically significant difference between the groups being compared. The statistical comparisons shown in Table 1 were performed using independent‐samples t‐tests for continuous variables and chi‐square (or Fisher's exact) tests for categorical variables, as described in the Statistical Analysis subsection of the manuscript.

### 3.2 Serum Olanzapine Concentration

**FIGURE 1:**
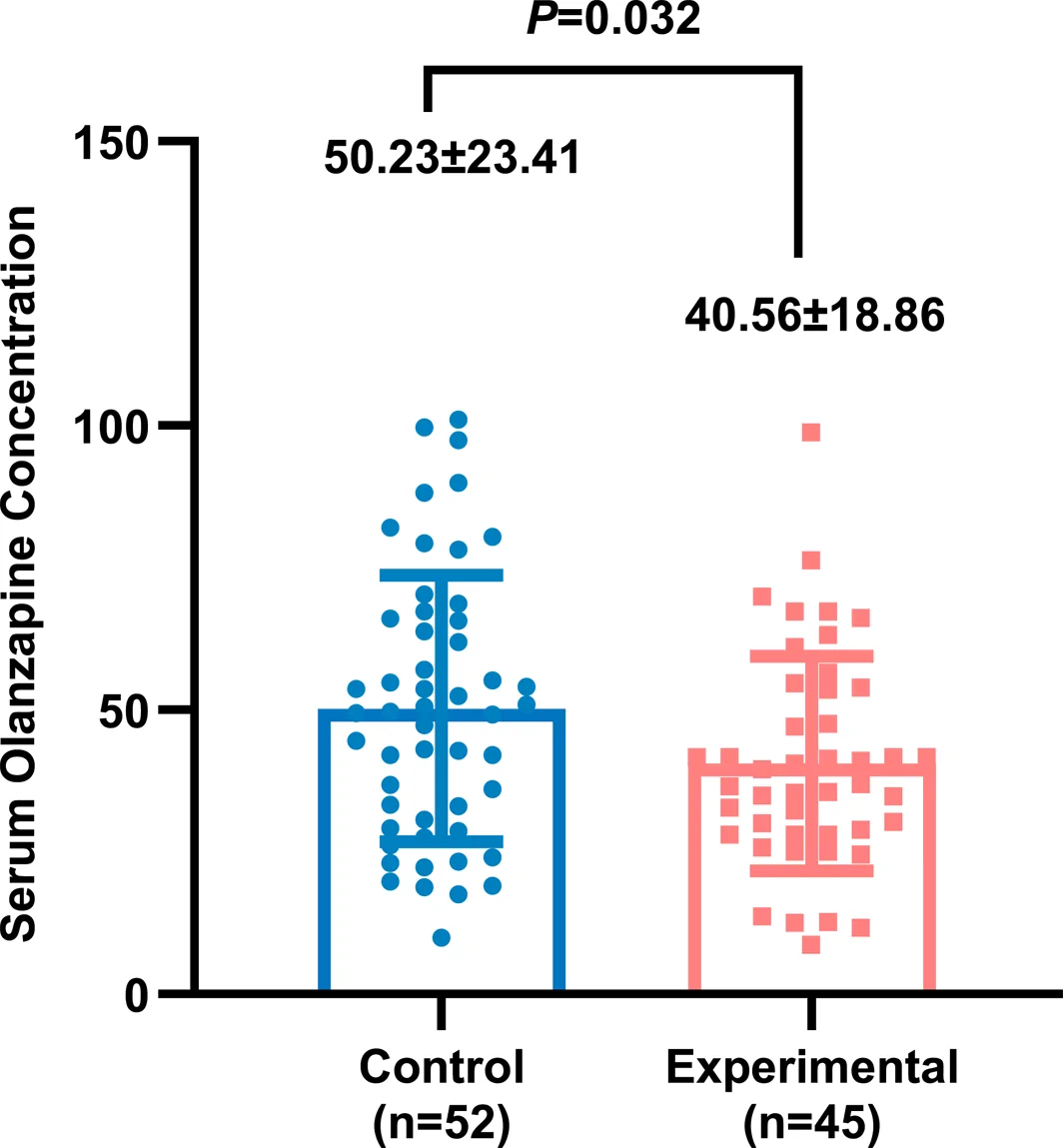
Comparison of serum olanzapine concentrations between the experimental group and the control group. Steady‐state serum olanzapine concentration in the experimental group (olanzapine + amlodipine) and the control group (olanzapine alone). Points represent individual participants, and bars and error bars represent the mean and standard deviation. The primary unadjusted Welch comparison yielded *p* = 0.027; the age‐adjusted ANCOVA sensitivity analysis yielded *p* = 0.032.

Mean steady‐state serum olanzapine concentration was lower in the combination group than in the olanzapine‐alone group (40.56 ± 18.86 vs. 50.23 ± 23.41 ng/mL) (Figure 1). The unadjusted mean difference (olanzapine alone minus combination) was 9.67 ng/mL (95% CI 1.15 to 18.20; Welch *p* = 0.027). In the age‐adjusted sensitivity analysis, the estimated difference was 9.80 ng/mL (95% CI 0.88 to 18.72; *p* = 0.032).

### 3.3 Clinical Efficacy

#### 3.3.1 PANSS Total Score Reduction

**FIGURE 2:**
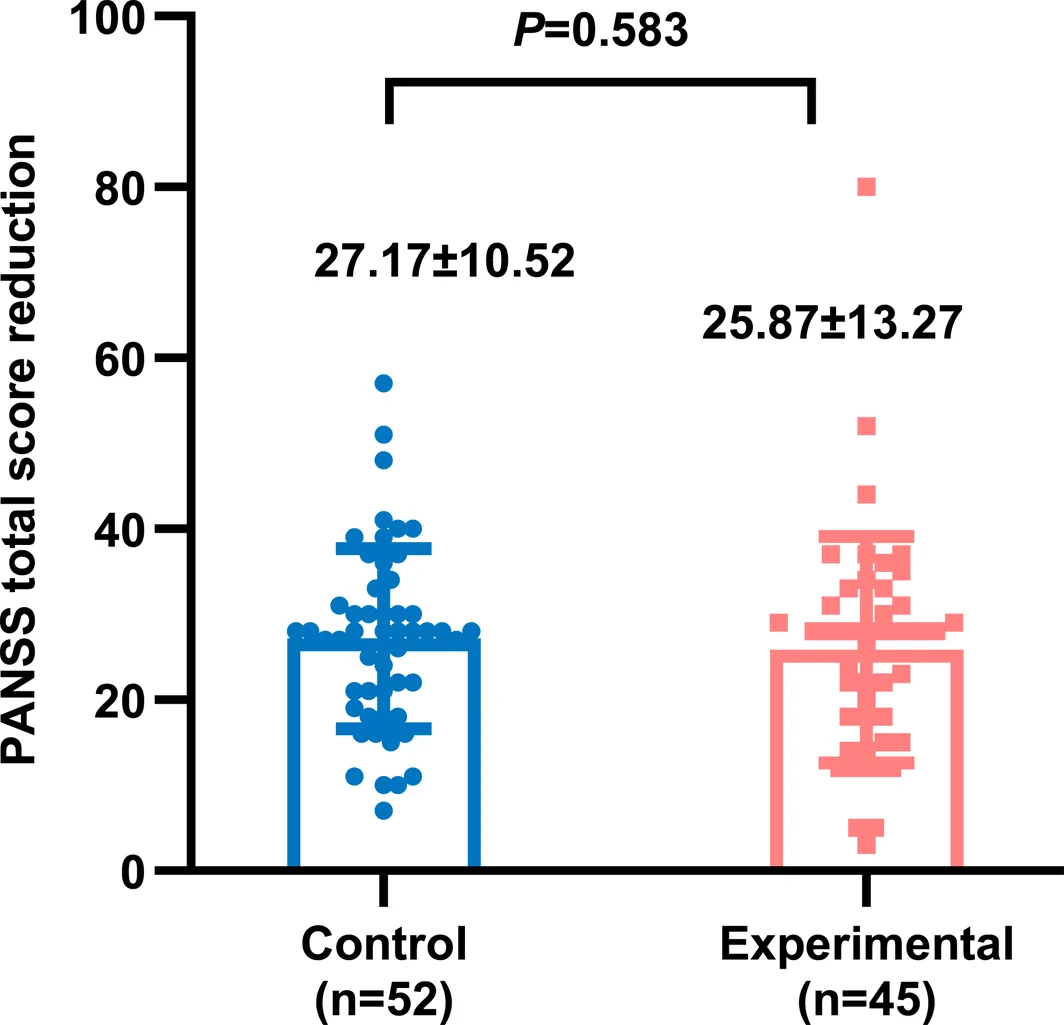
PANSS total score reduction from baseline to week 7 in the two groups. Points represent individual participants, and bars and error bars represent the mean and standard deviation. The between‐group difference was not statistically significant after adjustment for age and baseline PANSS total score *(p* = 0.583).

The analysis evaluated within‐patient change from baseline to week 7 rather than a cross‐sectional comparison. After adjustment for age and baseline PANSS total score, PANSS total reduction did not differ significantly between the combination group (25.87 ± 13.27) and the olanzapine‐alone group (27.17 ± 10.52; F (1,93) = 0.304, *p* = 0.583, partial *η* ^2^ = 0.003 (Figure 2)).

#### 3.3.2 PANSS Symptom‐Domain Reductions

**FIGURE 3:**
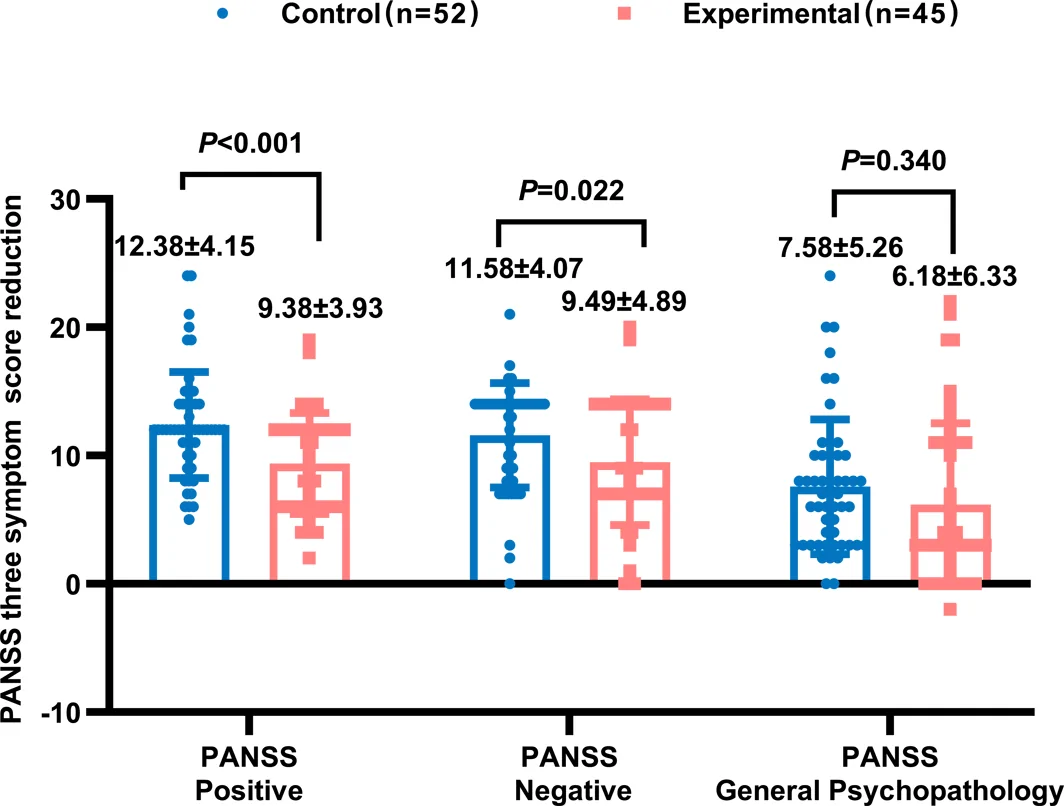
Changes in the recorded positive‐symptom, negative‐symptom, and general psychopathology domain scores from baseline to week 7. Points represent individual participants, and bars and error bars represent the mean and standard deviation. Between‐group comparisons were performed using ANCOVA adjusted for age and the corresponding baseline domain score. Improvements favoured the olanzapine‐alone group for the recorded positive‐symptom domain (*p* < 0.001) and negative‐symptom domain (*p* = 0.022), but not for the general psychopathology domain (*p* = 0.340).

Although PANSS total reduction was similar, adjusted improvements in the recorded positive‐ and negative‐symptom domains were smaller in the combination group (Figure 3).

Positive‐symptom domain reduction was 9.38 ± 3.93 in the combination group and 12.38 ± 4.15 in the olanzapine‐alone group. ANCOVA adjusted for age and baseline positive‐symptom score showed a significant group difference (F (1,93) = 21.032, *p* < 0.001, partial *η* ^2^ = 0.184).

Negative‐symptom domain reduction was 9.49 ± 4.89 in the combination group and 11.58 ± 4.07 in the olanzapine‐alone group. The adjusted difference remained significant (F (1,93) = 5.404, *p* = 0.022, partial *η* ^2^ = 0.055).

General psychopathology reduction was 6.18 ± 6.33 in the combination group and 7.58 ± 5.26 in the olanzapine‐alone group, with no significant adjusted difference (F(1,93) = 0.919, *p* = 0.340, partial *η* ^2^ = 0.010).

#### 3.3.3 SDSS Score Reduction

**FIGURE 4:**
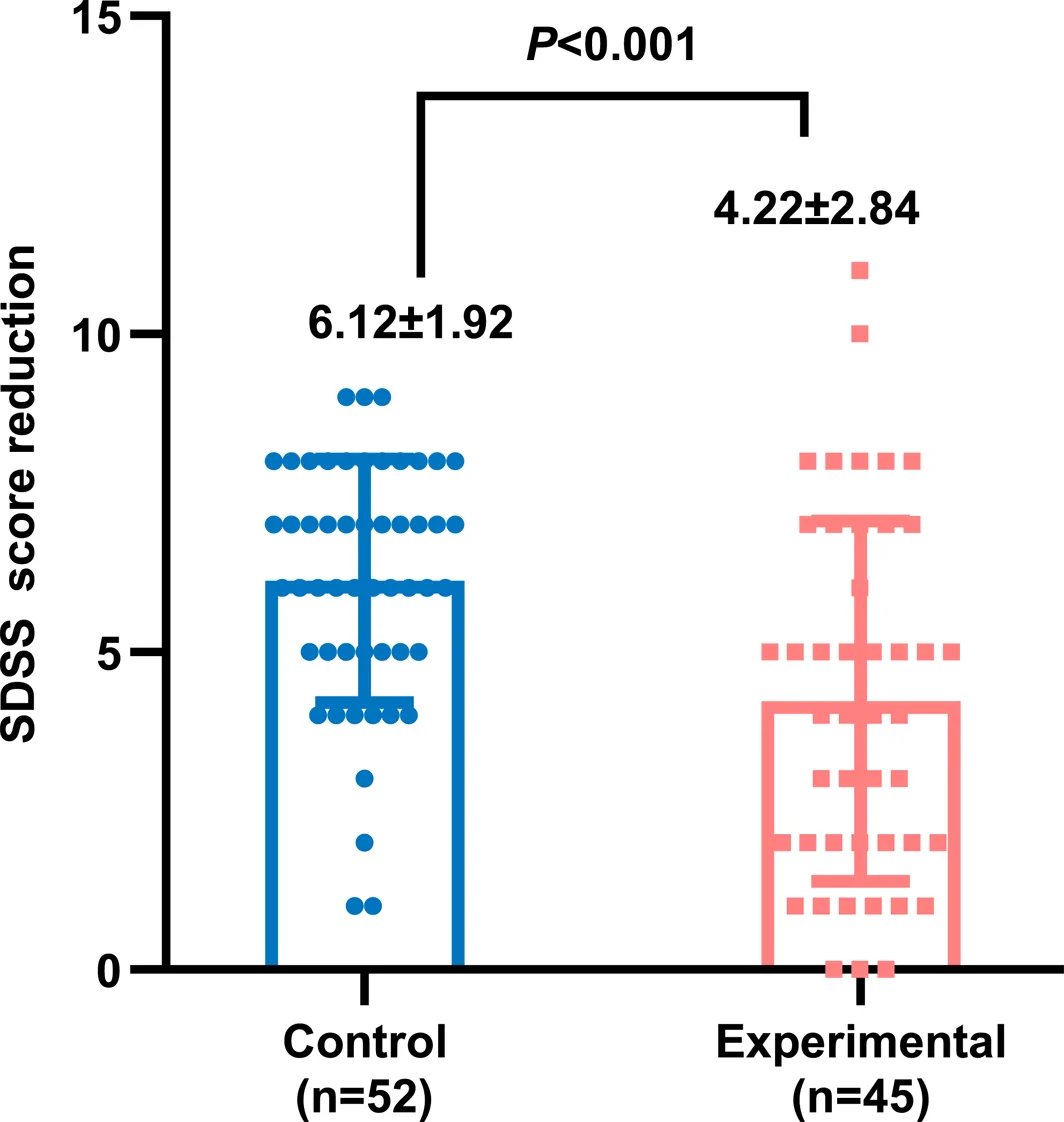
Change in SDSS score from baseline to week 7 in the control group (*n* = 52) and the experimental group (*n* = 45). Points represent individual participants, and bars and error bars represent the mean and standard deviation. Improvement was significantly smaller in the olanzapine‐plus‐amlodipine group after adjustment for age and baseline SDSS score (*p* < 0.001). A larger positive change indicates greater improvement.

SDSS improvement from baseline to week 7 was smaller in the combination group. After adjustment for age and baseline SDSS score, the reduction was 4.22 ± 2.84 in the combination group and 6.12 ± 1.92 in the olanzapine‐alone group (F (1,93) = 16.307, *p* < 0.001, partial *η* ^2^ = 0.149 (Figure 4)).

### 3.4 Association Between Serum Olanzapine Concentration and Clinical Score Changes

Spearman rank correlations were used because PANSS and SDSS change scores were non‐normally distributed. As shown in Table 2, serum olanzapine concentration was not significantly correlated with change in PANSS total score, positive‐symptom domain, negative‐symptom domain, general psychopathology domain, or SDSS score (all *p* > 0.05).

**TABLE 2:** Spearman rank correlations between steady‐state serum olanzapine concentration and changes in PANSS and SDSS scores from baseline to week 7.

Clinical outcome change	*n*	Spearman's *ρ*	*p* value
PANSS total score reduction	97	−0.116	0.258
Positive‐symptom domain score reduction	97	−0.044	0.672
Negative‐symptom domain score reduction	97	−0.131	0.202
General psychopathology domain score reduction	97	0.030	0.768
SDSS score reduction	97	0.089	0.387

*Note:* Change scores were calculated for each participant as the baseline score minus the week 7 score; therefore, a larger positive value indicates greater clinical improvement. Associations between steady‐state serum olanzapine concentration and changes in clinical scores were assessed using two‐sided Spearman rank correlation because the clinical score distributions were non‐normal. All analyses included 97 participants. No adjustment for multiple comparisons was applied because these analyses were exploratory. Abbreviations: PANSS, Positive and Negative Syndrome Scale; SDSS, Social Disability Screening Schedule.

Thus, although the combination group had both lower olanzapine concentrations and smaller improvements in selected clinical domains at the group level, the individual‐level analyses did not identify a detectable monotonic concentration‐response relationship.

### 3.5 Safety

**FIGURE 5:**
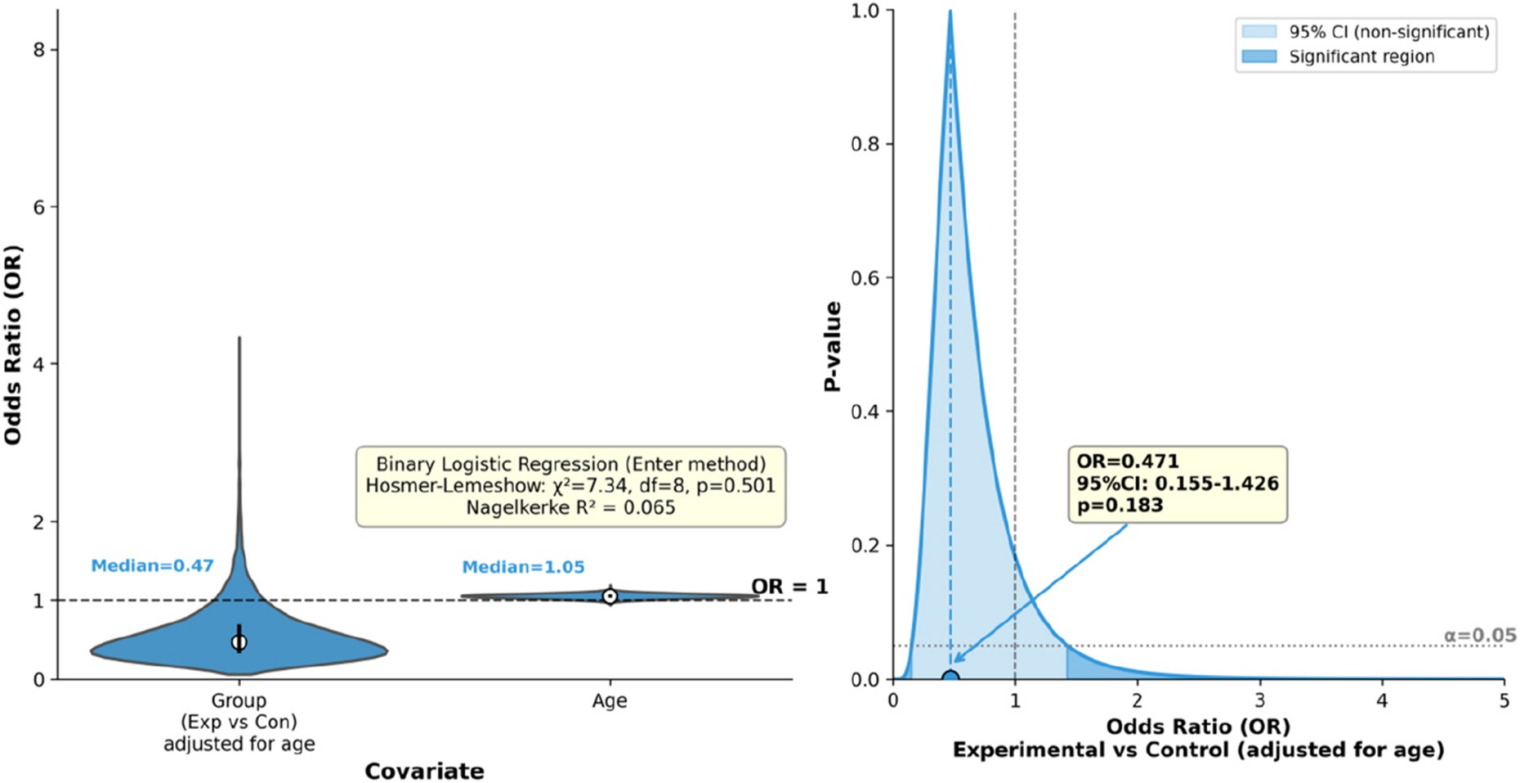
Adverse event rates (any RSESE‐recorded event) in the two groups. After adjusting for age, the difference was not significant (*p* = 0.183, logistic regression).

Any recorded adverse event occurred in 7/45 (15.56%) patients in the combination group and 12/52 (23.07%) in the olanzapine‐alone group (Figure 5). After adjustment for age, the difference was not statistically significant (OR = 0.471, 95% CI 0.155–1.426, *p* = 0.183). These data concern only the adverse events captured in the retrospective dataset; standardised longitudinal blood‐pressure measurements, orthostatic blood pressure, dizziness, syncope, and clinically significant hypotension were unavailable and could not be analysed.

## 4 Discussion

This retrospective study found that concomitant amlodipine use was associated with a lower mean steady‐state serum olanzapine concentration. The combination group also showed smaller improvements in selected symptom domains and social functioning, but not in PANSS total score or general psychopathology. Recorded adverse‐event rates did not differ. Importantly, individual serum olanzapine concentrations were not significantly associated with clinical change scores. These findings are exploratory and do not establish a causal pharmacokinetic or pharmacodynamic interaction.

### 4.1 Lower Olanzapine Concentration During Concomitant Amlodipine Use

Mean serum olanzapine concentration was approximately 19% lower in patients receiving concomitant amlodipine. Both group means remained within or close to recently proposed therapeutic reference ranges, although concentration‐response evidence remains heterogeneous and should be interpreted together with clinical response and tolerability (Hadjoudj et al. 2024; Wesner et al. 2023). Recent real‐world TDM studies further demonstrate that smoking, sex, body weight, hospitalization status, sampling time, and co‐medications can explain a clinically meaningful proportion of between‐patient variability (Ansermot et al. 2024; Bhavsar et al. 2024).

This finding may support considering therapeutic drug monitoring when clinical response is unexpectedly poor or when multiple factors affecting olanzapine exposure are present. However, the present observational data do not justify routine dose escalation or substitution of antihypertensive treatment solely on the basis of concomitant amlodipine use.

The mechanism underlying the observed concentration difference remains uncertain. Amlodipine is a weak CYP3A4 inhibitor, whereas CYP1A2 is an important oxidative pathway for olanzapine; weak CYP3A4 inhibition would not readily explain a lower olanzapine concentration (Kolli et al. 2023; Zhu et al. 2014). We found no direct evidence that amlodipine induces CYP1A2 or accelerates olanzapine clearance. Contemporary studies and reviews show that smoking, sex, body weight, sampling time, valproate use, genetic variation, and other co‐medications may materially affect olanzapine exposure (Ansermot et al. 2024; Beunk et al. 2024; Horvat et al. 2023; Zanni et al. 2025; Zhang et al. 2024). Differences in hypertension status, adherence, prior antipsychotic exposure, concomitant medications, or other unmeasured factors may therefore have contributed.

### 4.2 Symptom‐Domain Findings

The combination group showed smaller adjusted improvements in positive‐ and negative‐symptom domains, whereas PANSS total and general psychopathology changes were similar. These secondary findings should be interpreted cautiously because several clinical outcomes were tested without multiplicity adjustment and the retrospective design did not separate the effects of hypertension from those of amlodipine.

The observed domain‐specific differences do not establish a calcium‐channel‐mediated pharmacodynamic mechanism. Although calcium signalling is relevant to neuropsychiatric biology, the present study did not measure central target engagement, receptor occupancy, or pharmacodynamic biomarkers. Mechanistic explanations should therefore remain hypothesis‐generating.

The CATIE‐Sz analysis by Van Dyke and Thomas (2018) similarly found no significant difference in PANSS total scores during concomitant calcium‐channel blocker use, although the exposure definitions and clinical populations differed. The present findings extend that literature by reporting symptom‐domain changes, but confirmation in prospectively designed studies is required.

### 4.3 General Psychopathology

General psychopathology improvement did not differ significantly between groups. Given the broad and heterogeneous content of this domain, the absence of a group difference should not be interpreted as evidence for a specific receptor‐level explanation. It may instead reflect limited power, variability in symptom composition, or residual confounding.

### 4.4 Social Functioning and Concentration‐Response Analysis

SDSS improvement was smaller in the combination group. Because social functioning is influenced by symptoms, cognition, physical comorbidity, and social context, this association cannot be attributed specifically to amlodipine or to lower olanzapine exposure.

Importantly, steady‐state serum olanzapine concentration was not significantly correlated with change in any PANSS or SDSS outcome. Thus, although the combination group showed both lower concentrations and smaller improvements in selected domains, the individual‐level analyses did not identify a detectable concentration‐response relationship. These findings do not support directly attributing the clinical differences to lower olanzapine exposure. Hypertension, comorbid illness, concomitant medications, adherence, and other residual confounding may have contributed.

### 4.5 Safety and Haemodynamic Outcomes

Recorded adverse‐event rates did not differ significantly between groups. This result should not be interpreted as evidence that the combination has no haemodynamic risk. Additive blood‐pressure lowering is the best‐recognised potential interaction, but complete standardised baseline and follow‐up blood‐pressure measurements were not available. Orthostatic measurements and hypotension‐related symptoms were also not recorded systematically. Contemporary cardiovascular guidance for severe mental illness emphasizes proactive, multidisciplinary assessment of blood pressure and other cardiometabolic risk factors (Murphy and Daumit 2023; Polcwiartek et al. 2024). The present study therefore could not evaluate haemodynamic safety.

### 4.6 Limitations

Several limitations must be acknowledged. First, the non‐randomised groups differed simultaneously in hypertension status and amlodipine exposure; consequently, the effects of hypertension and amlodipine cannot be separated. Second, illness duration, prior antipsychotic exposure, adherence, smoking intensity, and comprehensive concomitant‐medication histories were incompletely documented. Third, the sample size was modest, and multiple exploratory clinical outcomes were tested without multiplicity correction. Fourth, amlodipine concentrations and relevant pharmacogenetic variables were not measured. Although the laboratory performance verification covered linearity, precision, recovery, lower limit of quantification, carryover, interference, matrix effects, and dilution integrity, stability testing was not included in the available report. Fifth, adverse events were captured only as a binary variable, and complete standardised blood‐pressure and hypotension data could not be analysed. Finally, the short follow‐up and single‐centre setting limit generalisability.

Prospective studies should enrol clinically comparable groups, document all concomitant medications and adherence, measure both olanzapine and amlodipine concentrations, and collect standardised seated and orthostatic blood pressure together with dizziness, syncope, and clinically significant hypotension. Such studies are required before causal or treatment‐adjustment recommendations can be made.

## 5 Conclusions

Concomitant amlodipine use was associated with lower steady‐state serum olanzapine concentration and smaller improvements in positive‐symptom, negative‐symptom, and social‐functioning scores in this retrospective cohort. PANSS total and general psychopathology changes were similar, and individual serum olanzapine concentrations were not correlated with clinical improvement. These hypothesis‐generating findings do not establish that amlodipine caused the concentration or clinical differences. Prospective studies with standardised blood‐pressure assessment and stronger control of confounding are needed.

## Author Contributions

**Xiaoxiao Chen:** conceptualization, writing – original draft. **Jing Ma:** writing – original draft. **Xiaolei Xuan:** formal analysis. **Mao Huang:** formal analysis, writing – review and editing. **Xue Yang:** writing – review and editing. **Baorong Huang:** conceptualization, writing – review and editing.

## Funding

This work was supported by the Joint Fund Project of Hubei Provincial Key Laboratory of Occupational Hazard Identification and Control, Wuhan University of Science and Technology (Grant No. JF2024‐G15).

## Ethics Statement

This study was approved by the Ethics Committee of Wuhan Wuchang Hospital (approval No. 2025‐001‐04) and was conducted in accordance with the Declaration of Helsinki.

## Consent

All named co‐authors consent to publication. All authors have accepted responsibility for the entire content of this manuscript and consented to its submission to the journal.

## Conflicts of Interest

The authors declare no conflicts of interest.

## Supporting information


Supporting Information S1

## Data Availability

The data that support the findings of this study are available from the corresponding author upon reasonable request.
