# Supplementary material for: Association of Concomitant Amlodipine Use With Serum Olanzapine Concentrations and Clinical Outcomes in Patients With Schizophrenia
Source: Hum Psychopharmacol. 2026 Sep 11;41(5):e70062. doi: 10.1002/hup.70062 (PMC13563104; doi:10.1002/hup.70062)
Supplement: Supplementary file 1 [file HUP-41-e70062-s001.docx]

**Supporting Information S1**

**Laboratory performance verification of the LC-MS/MS assay for serum olanzapine**

**S1. Analytical platform and scope**

Serum olanzapine concentrations were measured using an AB Sciex 4500MD liquid chromatography-tandem mass spectrometry system. Sample preparation, solvent preparation, calibration, and quality-control procedures followed the commercial reagent instructions used by the laboratory. The performance verification evaluated linearity, precision, recovery, lower limit of quantification, carryover, interference, matrix effects, and dilution integrity. The full laboratory report covered a multianalyte panel; only the olanzapine results are presented here.

**S2. Validation procedures**

Linearity was assessed at seven concentrations (5, 10, 20, 50, 100, 200, and 300 ng/mL), each measured in triplicate. Precision was evaluated at low and high concentrations using five replicate measurements in each of three analytical batches. Recovery was assessed using low- and high-concentration spiked serum samples measured in triplicate. The lower limit of quantification was evaluated using five replicate measurements at 5 ng/mL. Carryover was assessed by injecting double-blank samples after the highest calibrator. Interference was examined in three serum samples after addition of haemoglobin (2 g/L), bilirubin (342 micromol/L), or triglycerides (approximately 37 mmol/L). Matrix effects were assessed in five independent serum sources at low and high concentrations using internal-standard-normalised matrix factors. Dilution integrity was assessed after ten-fold dilution of a sample above the analytical measurement range.

**Table S1. Summary of olanzapine assay performance**

| **Performance characteristic** | **Acceptance criterion** | **Observed result** | **Conclusion** |
| --- | --- | --- | --- |
| Analytical measurement range | r >= 0.99; LLOQ bias <= 20%; other calibrator bias <= 15% | 5-300 ng/mL; r = 0.9988; calibrator bias -4.83% to 2.83% | Passed |
| Within-run precision | CV <= 15% | Low: 2.16%-6.58%; high: 2.53%-4.14% | Passed |
| Between-run precision | CV <= 15% | Low: 3.44%; high: 3.40% | Passed |
| Recovery | 85%-115% | Low: 100.7%; high: 107.4% | Passed |
| Lower limit of quantification | CV <= 20%; \|bias\| <= 20% | 5 ng/mL; CV 4.10%; \|bias\| 7.56% | Passed |
| Carryover | Analyte <= 20% of LLOQ peak area; internal standard <= 5% | Analyte 0.99%; internal standard 0.00% | Passed |
| Interference | \|bias\| <= 15% | All haemoglobin, bilirubin, and triglyceride biases within -4.90% to 8.22% | Passed |
| Matrix effect | Internal-standard-normalised matrix factor 0.8-1.2 | Low 0.851-1.100; high 0.830-0.912 | Passed |
| Ten-fold dilution integrity | Accuracy 80%-120% | 109.85% (bias 9.85%) | Passed |
| Clinical reportable range | Derived from LLOQ and validated dilution | 5-3000 ng/mL | Verified |

**Table S2. Linearity of the olanzapine assay**

| **Calibrator** | **Target (ng/mL)** | **Mean measured (ng/mL)** | **Bias (%)** | **Regression equation** | **Correlation coefficient** |
| --- | --- | --- | --- | --- | --- |
| J7 | 5 | 5.0 | -0.80 | y = 0.00338x + 0.00106 | r = 0.9988 |
| J6 | 10 | 10.1 | 1.23 |  |  |
| J5 | 20 | 20.3 | 1.67 |  |  |
| J4 | 50 | 48.8 | -2.33 |  |  |
| J3 | 100 | 102.8 | 2.83 |  |  |
| J2 | 200 | 190.3 | -4.83 |  |  |
| J1 | 300 | 308.0 | 2.67 |  |  |

**Table S3. Precision of the olanzapine assay**

| **Concentration level** | **Analytical batch** | **Mean (ng/mL)** | **Within-run CV (%)** | **Between-run CV (%)** |
| --- | --- | --- | --- | --- |
| Low | 1 | 14.84 | 2.16 | 3.44 |
| Low | 2 | 14.64 | 6.58 |  |
| Low | 3 | 15.62 | 2.62 |  |
| High | 1 | 239.2 | 3.48 | 3.40 |
| High | 2 | 236.6 | 4.14 |  |
| High | 3 | 252.0 | 2.53 |  |

**Table S4. Accuracy, LLOQ, and carryover**

| **Assessment** | **Level/target** | **Replicate results** | **Summary result** | **Acceptance criterion** |
| --- | --- | --- | --- | --- |
| Recovery | Low, 15 ng/mL | 15.3, 15.5, 14.5 ng/mL | 100.7% | 85%-115% |
| Recovery | High, 240 ng/mL | 269, 243, 261 ng/mL | 107.4% | 85%-115% |
| LLOQ | 5 ng/mL | 5.20, 5.59, 5.28, 5.18, 5.64 ng/mL | Mean 5.378 ng/mL; CV 4.10%; \|bias\| 7.56% | CV <= 20%; \|bias\| <= 20% |
| Carryover | Double blank after high calibrator | Analyte peak area 143 vs 14,500 at LLOQ; internal standard 0 vs 814,000 | Analyte 0.99%; internal standard 0.00% | Analyte <= 20%; internal standard <= 5% |

**Table S5. Interference assessment for olanzapine**

| **Serum sample** | **Reference concentration (ng/mL)** | **Haemoglobin bias (%)** | **Bilirubin bias (%)** | **Triglyceride bias (%)** |
| --- | --- | --- | --- | --- |
| 1 | 67.30 | -4.90 | -4.90 | -4.16 |
| 2 | 21.90 | 8.22 | 2.74 | 5.94 |
| 3 | 11.80 | -4.24 | -0.85 | -4.24 |

Note. Interference concentrations were haemoglobin 2 g/L, bilirubin 342 micromol/L, and triglycerides approximately 37 mmol/L. The acceptance criterion was an absolute bias no greater than 15%.

**Table S6. Matrix effects and dilution integrity**

| **Assessment** | **Sample/level** | **Observed result** | **Acceptance criterion** |
| --- | --- | --- | --- |
| Internal-standard-normalised matrix factor | Serum source 1 | Low 0.899; high 0.912 | 0.8-1.2 |
| Internal-standard-normalised matrix factor | Serum source 2 | Low 0.851; high 0.874 | 0.8-1.2 |
| Internal-standard-normalised matrix factor | Serum source 3 | Low 0.980; high 0.887 | 0.8-1.2 |
| Internal-standard-normalised matrix factor | Serum source 4 | Low 1.100; high 0.860 | 0.8-1.2 |
| Internal-standard-normalised matrix factor | Serum source 5 | Low 0.940; high 0.830 | 0.8-1.2 |
| Ten-fold dilution integrity | Sample above analytical range | Mean before multiplication 49.4 ng/mL; diluted result after factor 494 ng/mL; theoretical 450 ng/mL; bias 9.85% | Accuracy 80%-120% |
| Clinical reportable range | Olanzapine | 5-3000 ng/mL | LLOQ to validated 10-fold dilution upper limit |

**S3. Stability information**

Stability testing was not included in the available laboratory performance-verification report. No stability results were inferred or reconstructed. This limitation is stated in the revised manuscript and in the response to the reviewer.
